# Patterns of Rectal Temperature and Shipping Fever Incidence in Horses Transported Over Long-Distances

**DOI:** 10.3389/fvets.2019.00027

**Published:** 2019-02-14

**Authors:** Yousuke Maeda, Masa-aki Oikawa

**Affiliations:** ^1^Laboratory of Clinical Veterinary Medicine for Large Animal, School of Veterinary Medicine, Kitasato University, Towada, Japan; ^2^Diagnostic and Research Laboratory, Equine Veterinary Medical Center, Qatar Foundation, Doha, Qatar

**Keywords:** rectal temperature, long transport, incidence, shipping fever, horses

## Abstract

Shipping Fever is a transport associated syndrome seen in equids and bovines transported during long distances. The microbial profile and clinical signs vary between species, and in horses it is characterized by pharyngeal commensal bacteria and aerosolized particulate matter invading the lower airway due to compromised mucocillary clearance mechanisms during transports. This leads to pyrexia, pulmonary parenchymal disease, inappetence, and in severe cases pleuropneumonia. It has been shown that the incidence of transport-related pyrexia in horses increases with travel time and distance, however, this incidence rate has been expressed as the cumulative number of horses showing pyrexia with the length of travel time during road transport (cumulative percentage), which does not accurately reflect the actual temperature fluctuations and their patterns in relation to shipping fever. This study aims to demonstrate the individual fluctuations of body temperature variations during transport, particularly febrile changes. 53 Anglo-Arab and Thoroughbred horses aged 23–30 months were transported by road over different distances and durations (36–61 h; 1,492–2,921 km) in 3 investigations carried out in the spring and mid-summer in the northern hemisphere. The results showed that the incidence of fever (characterized by rectal temperature >38.6°C) was highest from 20 to 49 h after the start of transport. Clinical signs of shipping fever was observed in 25 of the 53 horses (47.2%), of which 10 horses (18.9%) exhibited fever at the end of transportation and 15 horses (28.3%) did not. This showed that horses that develop shipping fever do not necessarily present with fever at the end of transportation. Necropsy of 20 horses performed immediately after transportation suggested that transport induced pneumonia, contributed to the onset of pyrexia. This finding supports the suggestion that measuring body temperature upon arrival to determine the presence or absence of shipping fever could result in missed diagnoses for some horses with subclinical pneumonia, and that taking multiple temperature measurements at intervals from 20 h of transportation is a simple method for not missing horses with subclinical pneumonia.

## Introduction

Pneumonia associated with transport is one of the most common and detrimental sequelae of long distance transport of horses (1–4). This is characterized by microbial invasion of the lower airway, accompanied by environmental irritants including particulate hay, dust, carbon, and exhaust chemicals. Horses are commonly transported in close contact with horses of mixed origins, which leads to increased pathogen load among already compromised horses. In addition, horses are transported most commonly with the head maintained above the level of the withers. This is necessary for safety in many trailers, but causes significant compromise to the clearance mechanisms of the lower and middle airway, by preventing normal function of the mucocillary escalator, which relies on head positioning below the withers to adequately eliminate contaminants (5, 6). Horses in transit are subjected to increased challenge to the lower respiratory tract, and a significant number face away enough airway challenge to result in clinical signs that range from transient pyrexia and inflammatory infiltrate of the lower respiratory tract to fulminant parenchymal and pleural pneumonia (7, 8). A survey of shipping fever in Australia revealed an incidence rate of 9.2% in a survey (9), and another in Japan found a rate of 11.9% for horses transported for 25–28 h over distances of 1,000–1,300 km (10). In addition, one experimental study showed that transport-related pyrexia was not found when adult horses (mean age; 10.3 ±3.2 years) traveled for 94 h, including 51 h in transit and 43 h for rest stops at various points along the journey (11). The detrimental effects of transport associated pneumonia span from decreased performance upon arrival, to hypoxia and respiratory distress that requires humane euthanasia. (5, 6, 8, 10, 12–15). The initial clinical signs of shipping fever can be insidious, with the most common clinical sign being pyrexia (16). Several studies have shown that the incidence of transport associated pyrexia increases with travel time or distance (10, 14, 16). The incidence reported was based on cumulative percentage of horses showing pyrexia at different times during road transport (10, 14). The incidence was expressed as the percentage of affected cases accumulated as transport time progresses (the sum of all horses with pyrexia during transport) (10, 14). However, the actual number of affected horses fluctuates during transport (16, 17), because temperature can return to normal levels even if a fever develops at a certain point. As such, the total number of horses with fever may change over time (17), resulting in inaccurate estimates of actual percentages of transported horses being adversely affected by transport.

While there have been many studies investigating numerous aspects of shipping fever in horses, characterization of temperature alterations of horses while in transit has not been characterized. Most studies have focused on rectal temperature before and after transport (14, 16, 17) or for up to 7 days after cessation of transportation (11). However, a previous report by authors demonstrated cases in which horses that developed a fever during extended road transport returned to normal body temperature and appeared healthy at arrival, despite having subclinical pneumonic lesions (17). This suggests that basing the assessment of the presence or absence of shipping fever pneumonia only on the body temperature at the end of transportation could result in cases of subclinical pneumonia being missed. Pyrexia and other clinical signs that are insidious at the initial stages of the shipping fever may be missed by drivers and owners and, consequently, the incidence of the shipping fever could be underestimated (18–20) even though it is common industry practice to monitor rectal temperatures before and after long distance transport. In this study, published and unpublished data from 3 studies (14, 16, 17) on body temperature and fever development in horses during transportation were combined and assessed to: (1) clarify how body temperature and pyrexia developed in horses over time during transportation; (2) statistically examine the precision and sensitivity (diagnostic power) of using body temperature upon arrival as an indicator for predicting fever during transport; and (3) evaluate the pathological significance of transport-related pyrexia by investigating the correlation between the presence or absence of pulmonary lesions in horses euthanized humanely for necropsy immediately after arrival (at the end of transportation) and the onset of pyrexia during transport.

## Materials and Methods

### Horses

The specimens used in this study were a total of 53 thoroughbreds and Anglo-Arabian horses, which the author has previously described in three prior reports. Unpublished and published data regarding these horses were rearranged and searched (14, 16, 17). Therefore, the 29 horses mentioned in Table 1, Experiment 1 are the same 29 horses from Oikawa et al. (14) and Oikawa et al. (16) in the references, the 8 horses in Experiment 2 are the same 8 horses from Oikawa et al. (16), and the 16 horses in Experiment 3 are 16 out of 20 horses from Oikawa et al. (17). This total of 53 horses consisted of 27 females and 26 males, and 18 Anglo-Arabian and 35 Thoroughbred, aged 23 to 30 months (mean 26 ±1.9 months). The 45 horses used in Experiments 1 and 3 did not have previous experience of long-road transportation by truck, and the 8 horses used in Experiment 2 had little previous experience of long-road transportation by truck, only experiencing it once or twice (Table 1). All were without any previous history of clinical respiratory disease. Furthermore, all horses were in good health as ascertained by clinical examination prior to transport. The diagnostic criteria for the health condition of the horses according to clinical examination prior to transport were based on (i) lack of abnormality upon auscultation of the horse's heart, chest, and abdomen, (ii) a rectal temperature of 38.5°C or below, (iii) no presence of coughing or nasal discharge, and (iv) no abnormalities in the horse's energy or appetite. During and after transport, the horses were treated in accordance with the guidelines for humane use of experimental animals set out by the Equine Research Institute of the Japan Racing Association.

**Table 1:** Outline of transport experiments.

**Experiment**	**Number**			**Sex**		**Age**	**Breed**		Travel time*	**Distance**	**Season**	**Environment**		**Ref**.
	**T**	**P**	**NP**	**M**	**F**	**(m)**	**Th**	**AA**	**(h)**	**(km)**		**inside truck**		
												**TE**	**H**	
												**(°C)**	**(%)**	
1	29	11	18	18	11	23–27	16	13	36–39	1676	Spring	6–21	45–88	(10)
2	8	3	5	2	6	27–29	3	5	40–60	1858–2921	Summer	24–34	58–85	(13)
3	16	10	6	6	10	30–40	13	3	30–40	1502	Spring	9–24	31–64	(14)

Ref, references cited; T, total number of horses; p, number of horses affected with pyrexia; NP, number of horses without pyrexia; M, male; F, female; m, month; Th, thoroughbred; AA, Anglo-Arabian; h, hour; km, kilometer; TE, ambient temperature; H, humidity;

* *travel time including the time of stop rest*.

### Experimental Protocol

**Figure 1:**
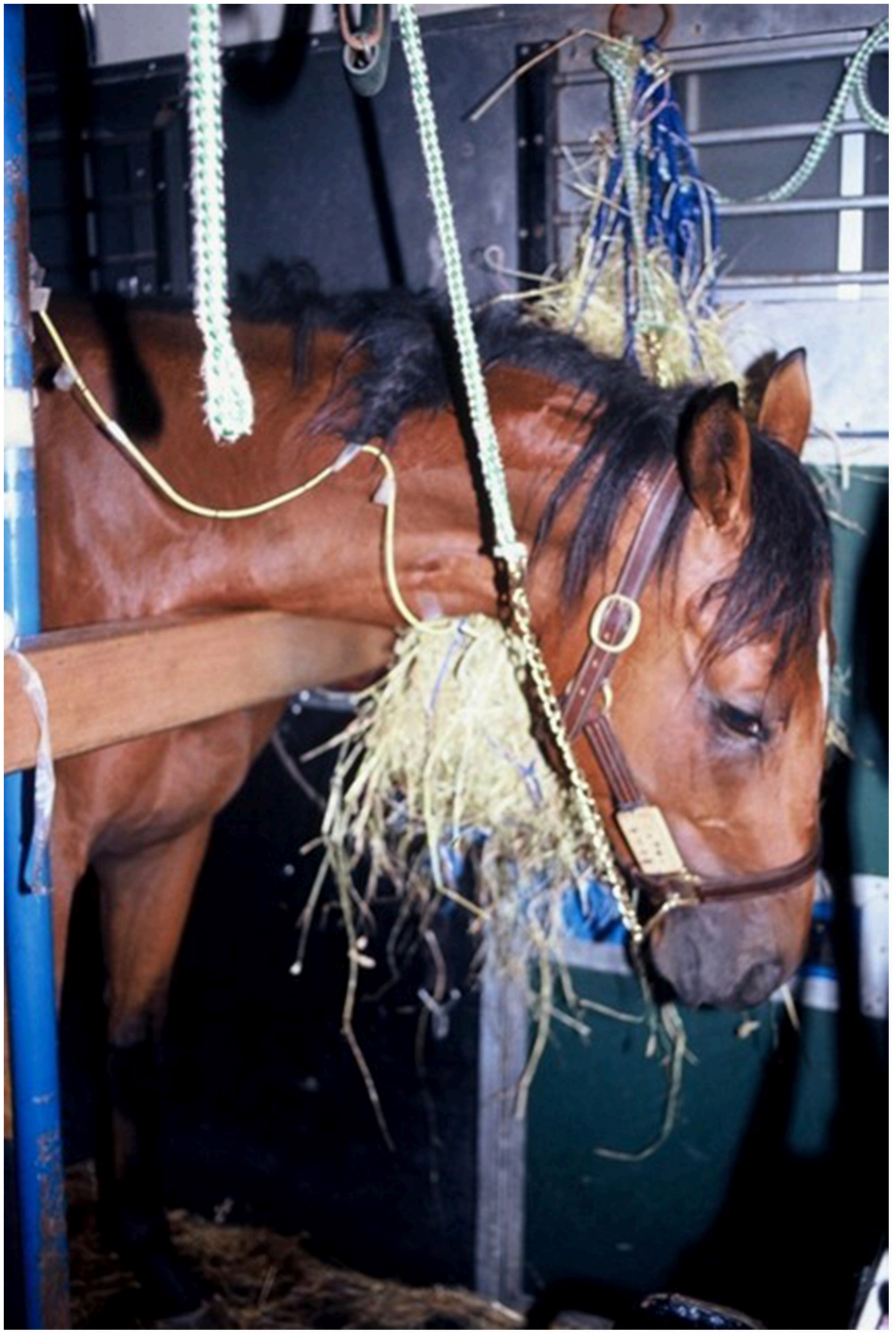
Horses restrained with a long tether. The head and neck turned downward below over a front wooden bar. Timothy hay was suspended in hay net close to nostril, i.e., the breathing zone.

**Figure 2:**
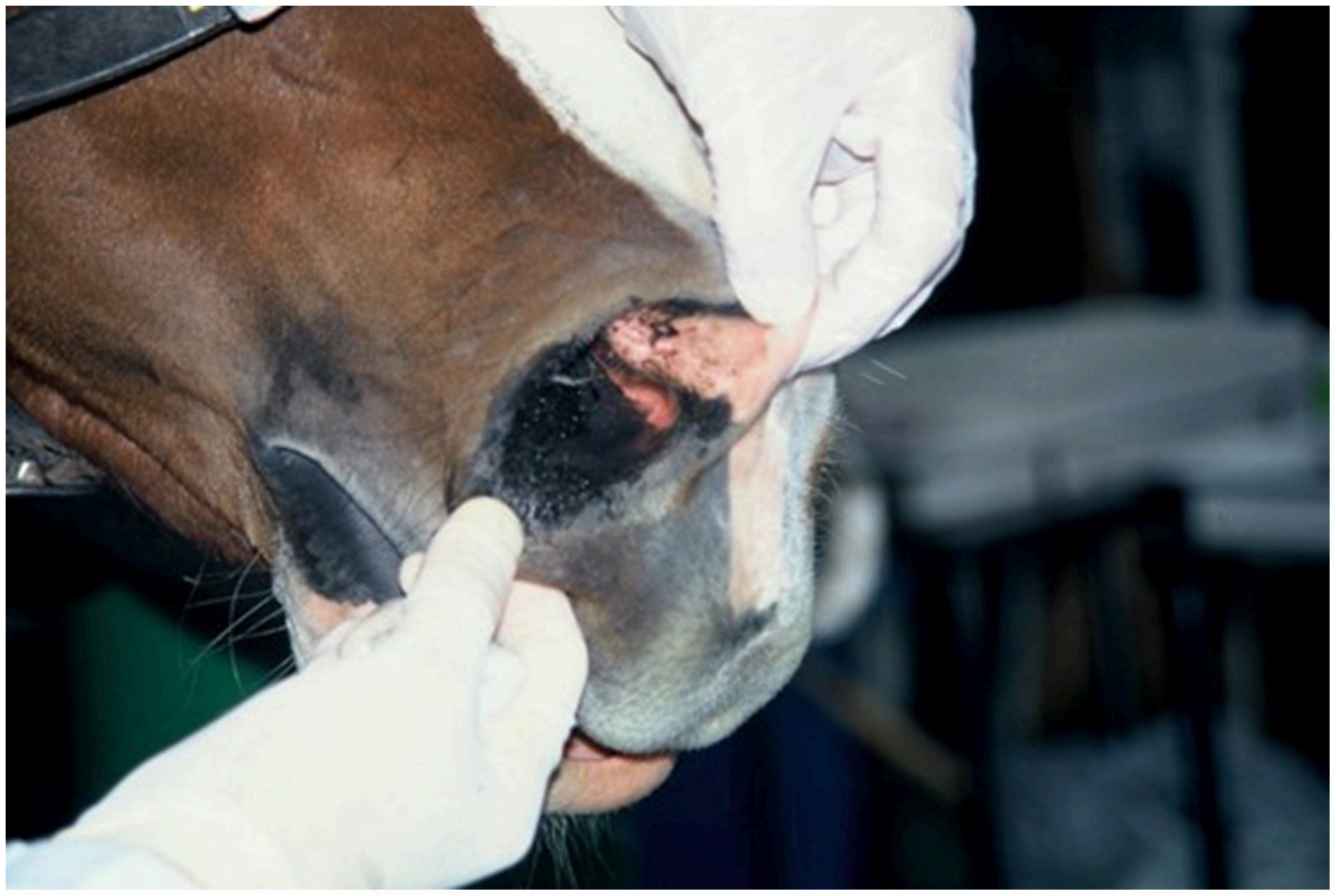
Nostril stained with mud from hay.

Three experiments of long-distance road transport by vehicle were conducted in April 1993 (Experiment 1) (14, 16), August 1993 (Experiment 2) (16), and September 1994 (Experiment 3) (17) (Table 1). Detailed experimental protocols of the transport methods in each experiment have been reported previously (14, 16, 17). Briefly, the horses the horses were loaded in groups of four or six into a 6 -horse capacity commercial vehicle (diesel trucks, Model KC-FW3FW, Hino Motors Ltd, Hino, Tokyo, Japan) and transported on the paved public road. The horses were separated by partitions, and untethered or restrained with a long tether to allow the horses to stretch their heads and necks downward below the point of shoulder height as well as side to side, but not able to lower their head below carpus height over a front wooden bar (height 1 meter). Each horse had ready access to a dry hay net suspended in front of its head throughout the journey (Figures 1, 2). Drinking water was available *ad libitum* throughout the experiments. Forty-nine horses were loaded into the trucks with their heads facing forward and 4 horses were loaded facing rearwards (Table 1). During the stop rest, the horses remained inside in their individual compartments and were not unloaded. The total travel time and distance were 36–60 h and 1,502–2,921 km, respectively (Table 1). Throughout the transport, following each 4–5 h period of transport, the horses were rested for 30 min to 1 h (Table 1). The trucks were driven by professional drivers working for commercial horse enterprise.

### Vehicle Interior Environment

During transport, changes in the ambient temperature and relative humidity were measured at 5 to 10 h intervals throughout the transport, while the vehicle was moving, as described previously (14, 16, 17).

### Monitoring of Rectal Temperature During Journey

Rectal temperature as well as assessments of the horse health were recorded by authors who rode with the horses in the vehicles throughout the journey. The criterion used to define transport-related pyrexia was a rectal temperature of more than 38. 6°C.

### Fluctuation of Rectal Temperature During Transportation

Rectal temperature data on 53 horses from 3 transportation experiments previously reported were used in the analysis. As much as possible, rectal temperature was measured during transportation at set intervals (approximately every 3–5 h) from immediately before the start of transportation to the end. However, it was difficult in practice to measure rectal temperature at exactly the same intervals. Therefore, to conveniently show the variation pattern of rectal temperature among the 53 horses as transport time progressed, the period from immediately before the start of transportation to the end was divided into 4-h intervals, then the number of horses that developed fever (>38.6°C) and the incidence of pyrexia were calculated for each segment.

### Ability to Predict Pyrexia Development From Rectal Temperature Upon Arrival

Receiver Operating Characteristic (ROC) curve analysis as a univariate analysis was performed to explore whether temperature at the end of transportation can be a potential clinical predictor of the onset of transport-related pyrexia (21–23). The conducted ROC curve analysis is described in the Statistical Analysis section.

### Pathological Examination

Twenty randomly selected horses with and without pyrexia were euthanized for necropsy immediately after transportation. Macroscopic inspections were performed with special attention to pneumonic lesions. To confirm the nature of pneumonic lesions, samples for histological, electron microscopic, and bacteriological examination were collected from pneumonic lesions as described previously (16, 17).

### Correlation Between Pyrexia and Pneumonic Lesions

To clarify the relationship between pyrexia and pneumonic lesions, 20 horses examined for necropsy were divided into a pyrexia group, non-pyrexia group, pneumonic lesion group, and non-pneumonic lesion group.

### Statistical Analysis

The statistical tests and subjects of testing are as indicated below.

- The significant difference in the incidence rate of pyrexia among the 3 groups that were transported in Experiments 1, 2, and 3 were compared using Pearson's chi-squared test (χ^2^).
- The highest temperature of the horses during transportation was compared using one-way repeated measures ANOVA among the 3 groups of horses in Experiment 1 (29 horses), Experiment 2 (8 horses), and Experiment 3 (16 horses).
- As shown in Table 2, the rectal temperature prior to departure (A) and upon arrival (B) for all 53 horses, prior to departure (C) and upon arrival (D) for the 25 horses with pyrexia, and prior to departure (E), and upon arrival (F) for the 28 horses without pyrexia, were compared using the chi-square test.
- Incidence rates of pyrexia were compared between designated segments as set intervals (approximately every 3–5 h) using the chi-square test.
- To explore whether the horses' temperature upon the completion of transportation could be a reliable marker to estimate the onset of transport-related pyrexia, a logistic regression analysis was performed to assess the correlation between the horses' rectal temperature on arrival with their temperatures obtained during transport, with the horses' temperature upon arrival as the independent variable (continuous variable) and the presence or absence of transport-related pyrexia (38.6°C) as the dependent variable (categorical variable). This was followed by a reliever operating characteristic (ROC) curve analysis to assess the performance (diagnostic accuracy) of the rectal temperature on arrival as the independent variable for the development of transport pyrexia. The cut-off value for the temperature upon arrival was above 38.6°C for the ROC curve analysis.
- The Fisher's exact test was used to calculate correlation coefficients between the necropsied horses with pneumonic lesions and the necropsied horses without pneumonic lesions, to assess relationship between development of pyrexia and presence or absence of pulmonary lesions.
- For all statistical analyses, a *P*-value of 0.05 was considered statistically significant.

**Table 2:** Rectal temperature prior to departure and upon arrival.

**Group**	**N**	**Mean ± SD**	**LL**	**UL**	**Statistical analyses**
**TOTAL (53 HEADS)**					
A (Rectal temperature prior to departure)	53	37.989 ± 0.147	37.4	38.4	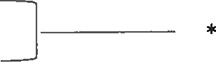
B (Rectal temperature upon arrival)	53	38.402 ± 0.493	37.6	40.0	
**HORSES WITH PYREXIA**					
C (Rectal temperature prior to departure)	25	37.984 ± 0.193	37.4	38.4	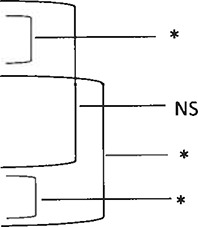
D (Rectal temperature upon arrival)	25	38.584 ± 0.638	37.6	40.0	
**HORSES WITHOUT PYREXIA**					
E (Rectal temperature prior to departure)	28	37.993 ± 0.088	37.8	38.2	
F (Rectal temperature upon arrival)	28	38.200 ± 0.277	37.6	38.5	

N, number of horses; SD, standard deviation; LL, lower limit of rectal temperature; UL, upper limit of rectal temperature; ^*^statistically significant of rectal temperature between groups; NS, not statistically significant.

## Results

### Vehicle Interior Environmental Changes

The internal truck air temperature and relative humidity during transport in the truck ranged from 6 to 21°C and 45–88%, respectively, in Experiment 1; 24 to 34°C and 58–85%, respectively, in Experiment 2; 9 to 24°C and 31–64%, respectively in Experiment 3 (Table 1). These temperatures and humidities inside vehicles were within the standard thermoneutral zones for the horses (24).

### Incidence Rate in Three Groups

The incidence rate of pyrexia among the three groups of horses during transportation in Experiments 1, 2, and 3 was 37.9% (11 out of 29 horses), 37.5% (3 out of 8 horses), and 62.5% (10 out of 16 horses), and results of the comparison using Pearson's chi-squared test (χ^2^) showed no significant difference among the various groups.

### Mean Value of Upper Limit of Rectal Temperature Among the Three Groups

The mean value of the highest temperature of the 53 horses during transportation was 38.68 ± 0.55 (*n* = 29), 38.87 ± 0.93 (*n* = 8), and 38.81 ± 0.51 (*n* = 16) in each group in Experiments 1, 2, and 3. No significant difference was found between each group after comparing these mean values using the one-way repeated measures ANOVA among the groups.

### Rectal Temperature Prior Transport and After Arrival

From immediately before the start of transportation to the end, pyrexia was observed in 25 horses (47.2%) of the 53 horses and was not observed in 28 horses (52.5%) (Table 2). Of the 25 horses that exhibited pyrexia, 10 horses (18.9%) exhibited fever at the end of transportation, while 15 horses (28.3%) did not. Table 2 shows the temperatures upon departure and at arrival for all 53 horses, including the 25 horses with pyrexia, and the 28 horses without pyrexia. Significant differences were observed between the departure and arrival temperatures for the all 53 horses, including the 25 horses with pyrexia, and the 28 horses without pyrexia (Table 2). There was not significant difference between the departure temperature for the horses with pyrexia and the horses without pyrexia. However, a significant difference was observed between the arrival temperatures in the horses with pyrexia and horses without pyrexia (Table 2).

### Fluctuation of Rectal Temperature During Transport

**Figure 3:**
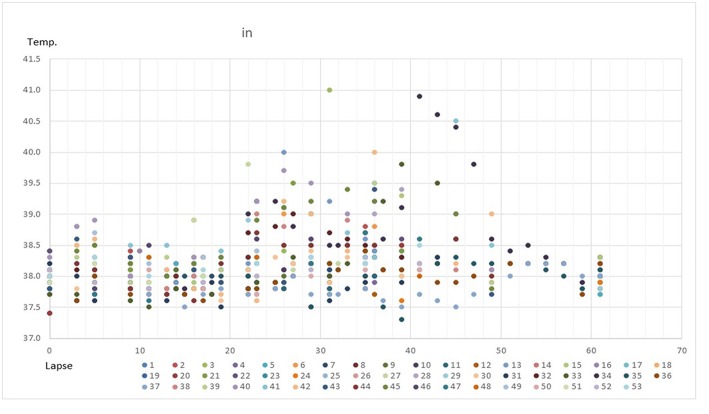
Fluctuation of rectal temperature in the 53 horses throughout transport. Horizontal and vertical axes indicate travel time (hour) and rectal temperature (°C), respectively.

Fluctuation of rectal temperature in the 53 horses throughout transport is shown in Figure 3. There was a tendency for the rectal temperature to increase over the travel time, regardless of duration of the journey.

### Incidence of Pyrexia Over the Duration of the Travel Time

Table 3 shows the number of horses with pyrexia at every the 4-h interval during transport. It should be noted, that while 53 horses were examined, not every horse was measured in each time segment and some horses were measured multiple times. Therefore, the total number measured was not 53 for each segment. The incidence rate of horses with pyrexia was higher in the segments between 20 and 49 h. In the other time segments, the incidence was low, at around 5%.

**Table 3:** Incidence of pyrexia over the duration of the travel time.

4-Hour interval (h)*	N**	**Number of horses with pyrexia**	**Incidence of horses with pyrexia (%)**
0–4	69	3	4.3
5–9	58	3	5.2
10–14	44	0	0
15–19	65	2	3.1
20–24	46	12	26.1
25–29	66	19	28.8
30–34	37	8	21.6
35–39	77	18	23.4
40–49	32	7	21.9
50–60	24	0	0

* 4-h interval from start of transportation to the end except 40–49 and 50–60 h;

** *Number of horses measured rectal temperature for each 4-h-segments*.

### Ability to Predict Pyrexia Development From Temperature Upon Arrival

**Figure 4:**
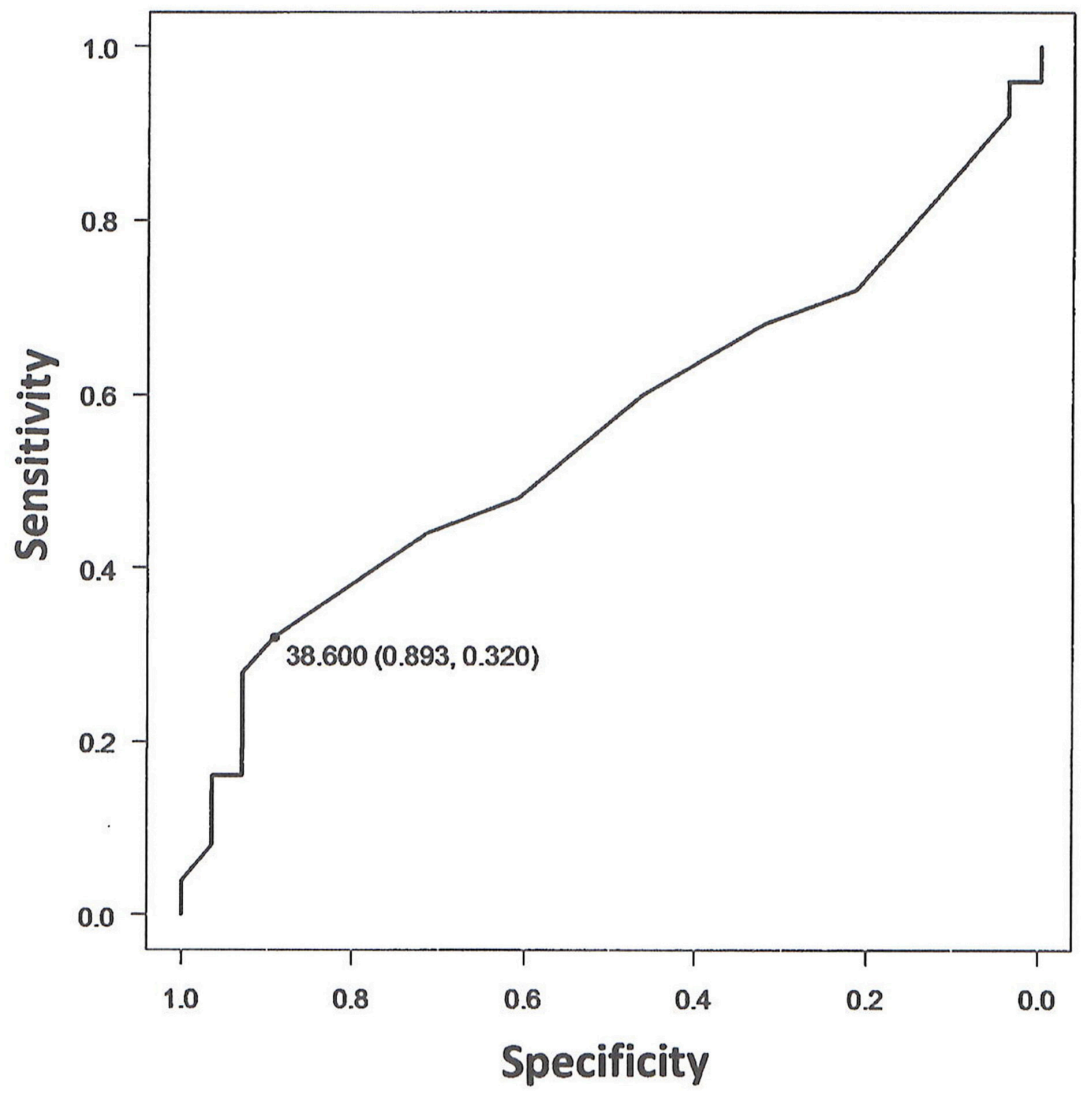
ROC curve displaying of sensitivity on y-axis and specificity on x-axis. The area under the curve (AUC) is a measure of sensitivity and specificity for assessing validity of diagnostic potential. The AUC closer to 1 indicates better performance of the clinical predictors in differentiating the presence or absence of transport-related pyrexia. The figure indicates that using 38.6°C as the cut-off value for the temperature upon arrival to diagnose the presence or absence of transport-related pyrexia had a specificity of 89.3% and sensitivity of 32.0%.

The results of the logistic regression analysis between the horses' rectal temperature on arrival and presence or absence of transport-related pyrexia (38.6°C) are indicated in Table 4. Specifically, the odds ratio of rectal temperature upon arrival of the 53 horses suggested that a 1°C increase in temperature upon arrival was associated with a 2.23-fold higher risk of shipping fever, however this result was not significant (Table 4). The area under the curve (AUC) from the ROC curve analysis was 0.56, which was not significant for diagnostic accuracy. Using 38.6°C as the cut-off value for temperature upon arrival to diagnose the presence or absence of transport-related pyrexia had a specificity of 89.3%, but a low sensitivity of 32.0% (Figure 4; Table 5).

**Table 4:** Logistic regression analysis of rectal temperature upon arrival as a marker for estimating pyrexia.

	**Logistic regression analysis**			
	**95% confidence interval**			
**Independent variable**	**Odds ratio**	**Lower limit**	**Upper limit**	***p*****-value**
Rectal temperature upon arrival (°C)	2.23	0.69	7.14	0.178

*Independent variable: rectal temperature upon arrival; Dependent variable: presence or absence of transport- related pyrexia*.

**Table 5:** Logistic analysis of rectal temperature upon arrival for estimating sensitivity and specificity as diagnostic marker of pyrexia.

**Independent variable**	**Receiver operating characteristics (ROC) curve analysis**					
	**95% Confidence interval**					
	**AUC**	**Lower limit**	**Upper limit**	***p*-value**	**Sensitivity (%)**	**Specificity (%)**
Rectal temperature upon arrival	0.56	0.40	0.72	0.454	32.0	89.3

*Independent variable: rectal temperature upon arrival; Dependent variable: presence or absence of transport-related pyrexia, AUC: area under the curve*.

### Distribution of Pneumonic Lesions and Their Pathological Features

**Figure 5:**
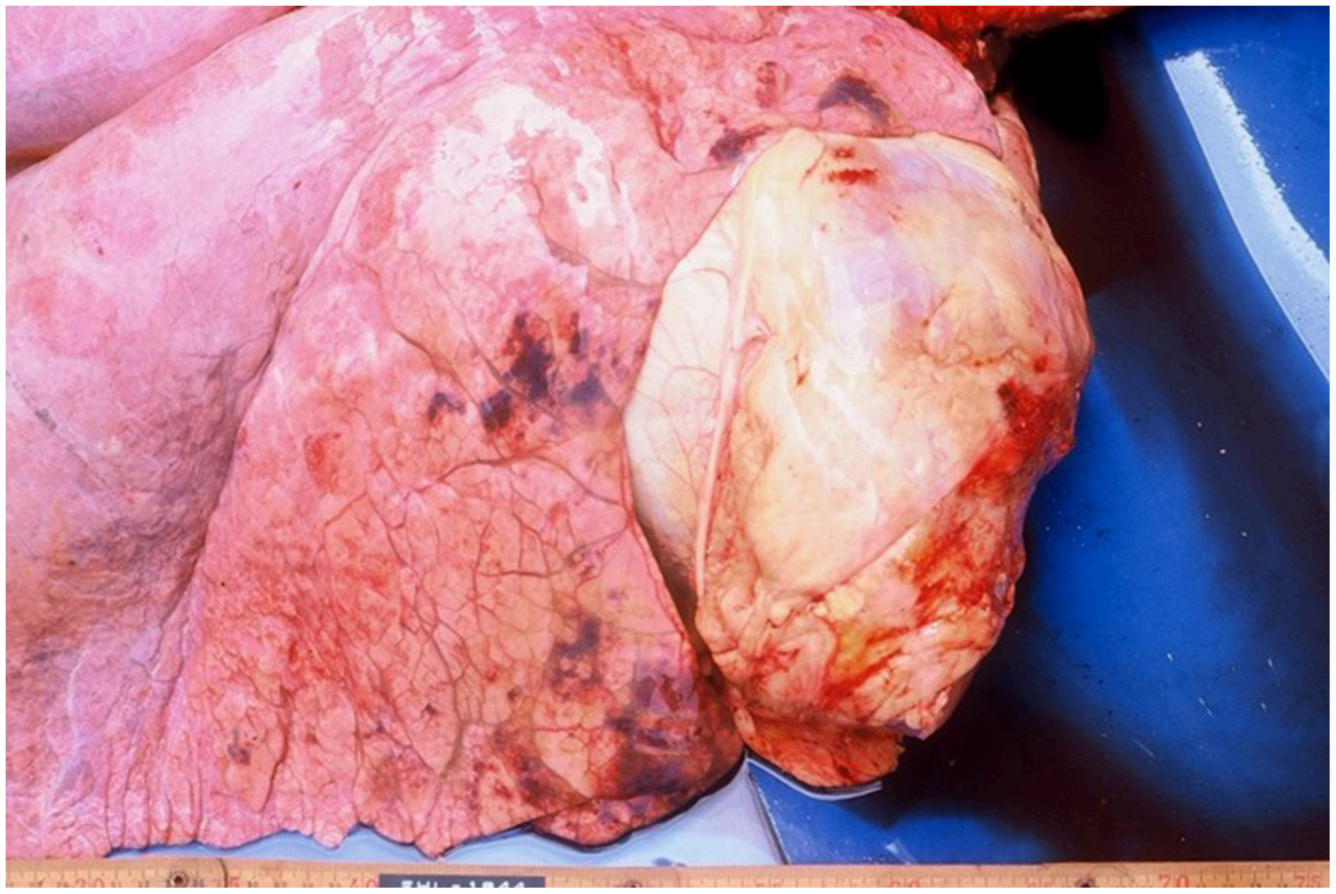
Macroscopic pneumonic lesions with dark red in color at the right cranial lung lobe and the cranial portion of the caudal lung lobe.

**Figure 6:**
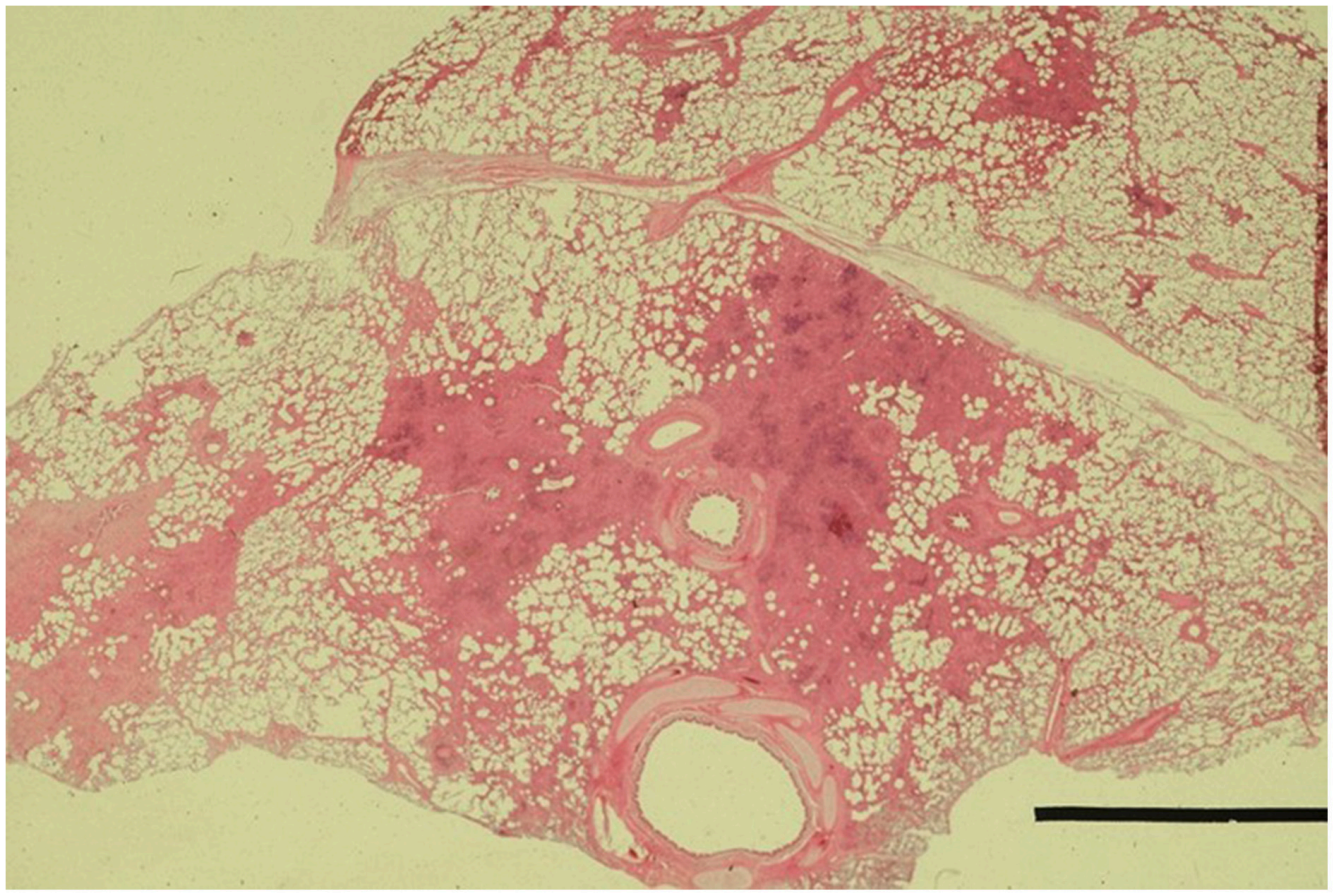
Small foci of bronchopneumonia in the pneumonic lesion. Hematoxylin and Eosin staining. Bar, 1 mm.

**Figure 7:**
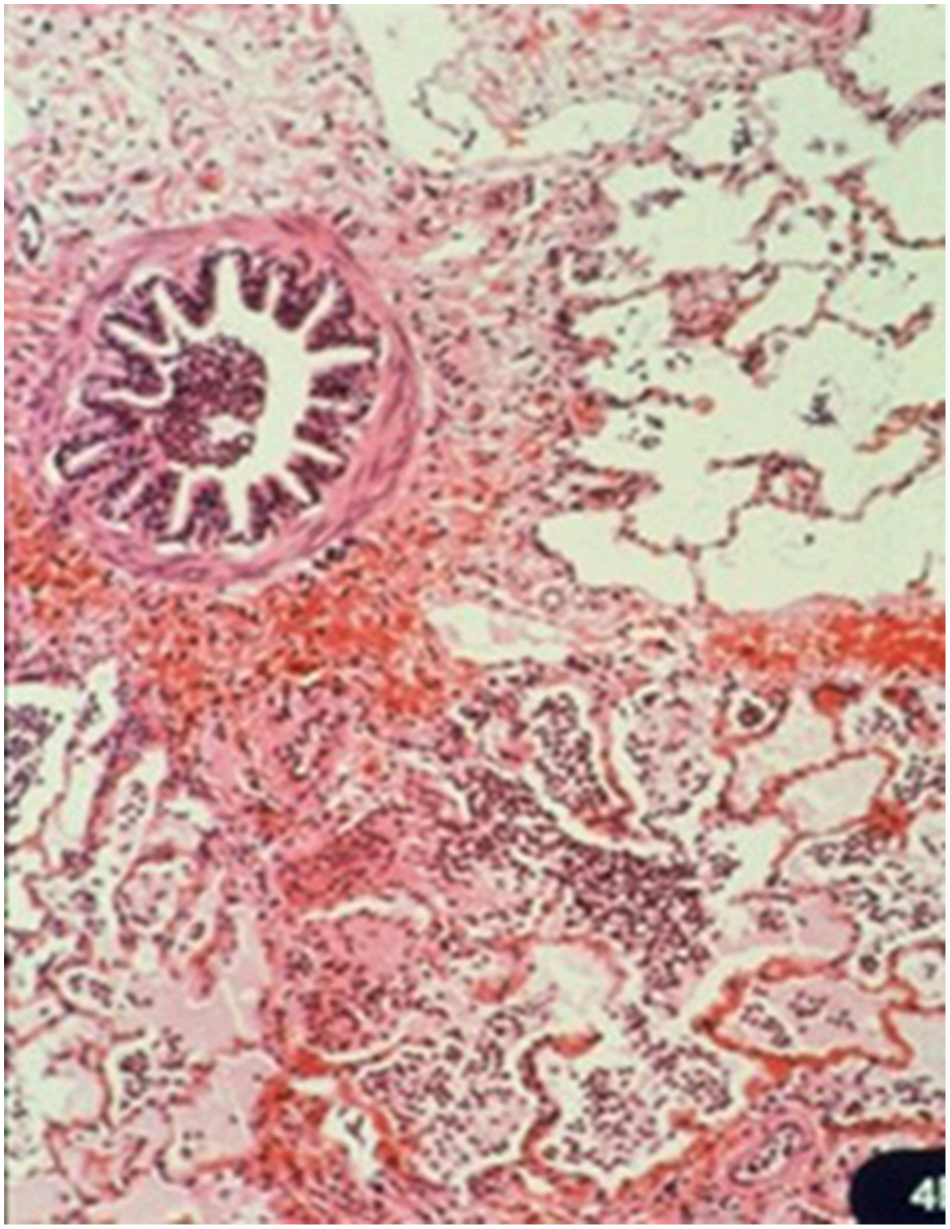
Acute serous neutrophilic bronchopneumonia. Bronchiolar and alveolar lumen containing numerous neutrophils, macrophages, desquamated epithelial cells, and fibrinous exudates. Hematoxylin and Eosin staining. × 124.

**Figure 8:**
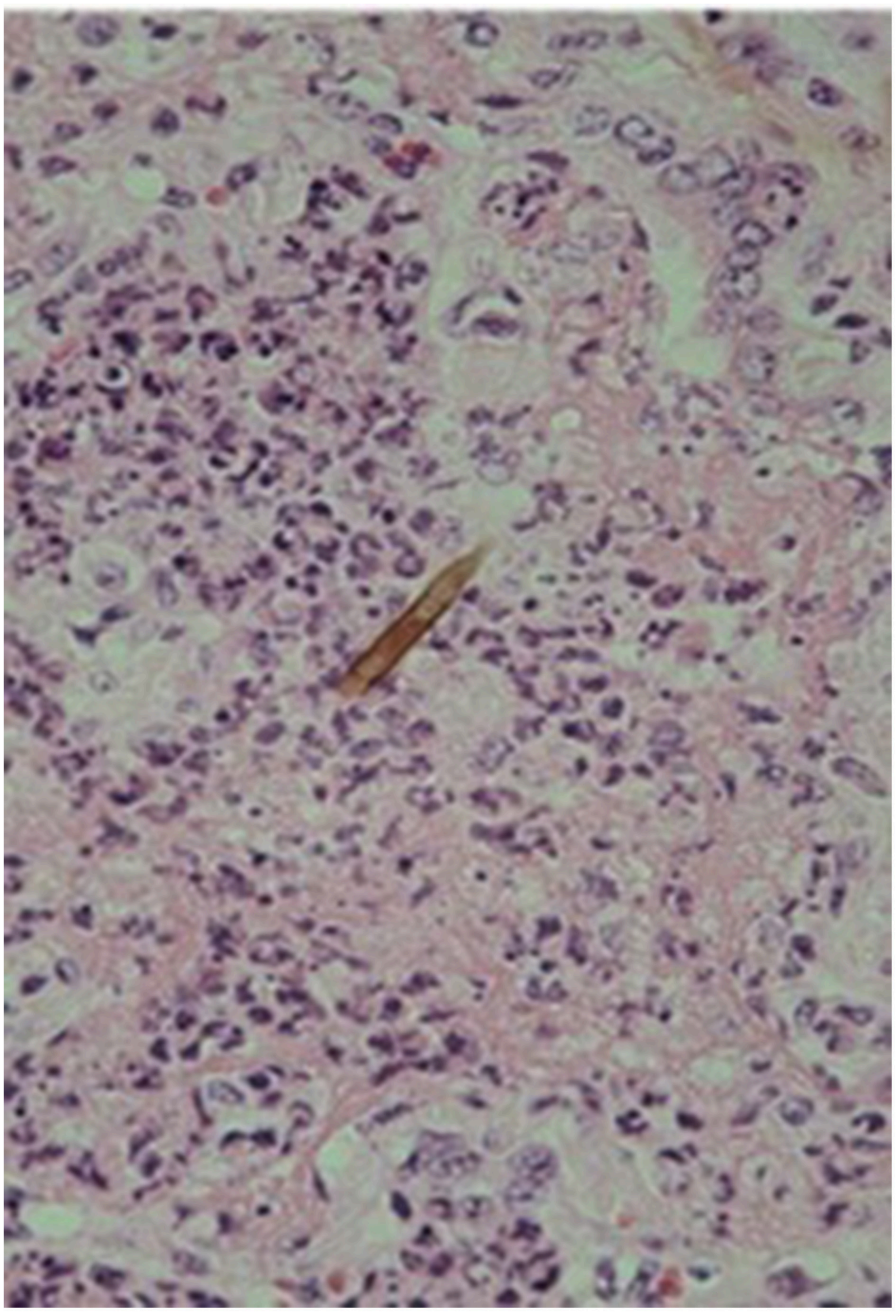
Particle of hay (arrowhead) in the alveolar lumen of serous neutrophilic bronchopneumonia. Hematoxylin and Eosin staining. × 180.

Macroscopically, small and well-defined dark red colored areas of consolidation were seen in 7 of the 20 horses necropsied (Figure 5). As shown in Table 6, most of these lesions were found in the cranioventral portion of the caudal lung lobe with a propensity to affect the right lung. A consistent pathological finding of the lesions was acute serous neutrophilic bronchopneumonia (Figures 6, 7). Numerous macrophages, neutrophils, desquamated epithelial cells, fibrinous exudates and particles of hay were observed in the bronchial, and bronchiolar lumen (bronchopneumonia) (Figure 8). The alveolar septa were thickened by hyperemia and infiltration of neutrophils (alveolar capillaritis).

**Table 6:** Distribution of pneumonic lesions in horses with pyrexia.

**Region of lung/Horses with pyrexia**	1^*^	2^*^	3^*^	4^*^	5^*^	6*	7^*^
**RIGHT LUNG**						
Cranial lobe	+						
Craniodorsal portion of caudal lobe							
Cranioventral portion pf caudal lobe	+	+	+	+	+	+	+
Caudodorsal portion of caudal lobe							
Caudodorsal portion of caudal lobe							
Caudoventral portion of caudal lobe		+					
Accessory lobe	+						
Left lung							
Cranioventral portion of caudal lobe						+	

* *Horses with pyrexia; +, pneumonic lesion*.

### Bacteriology

Aerobic and anaerobic bacteriological culture of the pneumonic lesions revealed a mixed population of bacteria with the predominant organisms, being *Streptococcus* spp., *Pasteurella* spp., *Staphylococcus* spp., *Bacteroides* spp., and *Enterococcus* spp*. Streptococcus equi* subsp. *zooepidemicus* antigen was found in the cytoplasm of neutrophils and macrophages in the alveolar lumen.

### Correlation Between Pyrexia and Pneumonic Lesions

Table 7 shows correlation between pyrexia and pneumonic lesions in 20 cases necropsied. Data were analyzed by Fisher's exact test. A significant result was obtained (*p* < 0.001), indicating a positive correlation between the group with pyrexia and the presence of pneumonic lesions.

**Table 7:** Correlation between pyrexia and pneumonic lesions in 20 cases necropsied.

	**Number of horses with pyrexia**	**Number of horses without pyrexia**	**Total number**
Horses with pneumonic lesions	6 (2.1)*	1 (4.9)	7
Horses without pneumonic lesions	0 (3.9)	13 (9.1)	13
Total number	6	14	20

*The figures in parentheses indicate the expectation values*.

* *Statistically significant between the group with pyrexia and the presence of pneumonic lesions*.

## Discussion

Based on the European and Australian Code of Animal Transportation (25, 26), the shipping scenarios used in this analysis were categorized as a long journey (>24 h). The transport conditions differed in terms of when the journey took place (season), the length of travel time and distance, route, and environmental factors such as the interior air temperature and humidity of the vehicle. Moreover, the 53 horses had different horse-related factors (individual differences), such as temperament, the level of travel experience, age, sex etc., and included both well-trained/conditioned performance horses and experimental horses that had not been acclimatized to long distance transport. In contrast to the shipping fever incidences of 9–12% determined in previous studies (9, 10), the average of incidence of transport-related pyrexia in the present analysis is 47.2% as mentioned above. This may reflect differences in the travel durations or the experience level of the horses in long travel times. In our study, the travel experience was low because most of the horses used in the experiments were young (mean age; 26 months) and inexperienced horses. The way of restraining the horses in a head-up posture which was not able to lower their head below carpus height over a front wooden bar during long-term transport used in the present experiments might have led to a bacterial invasion of the lower airway by oral-pharyngeal commensal organisms acting as opportunistic pathogens of the lower airway, thus leading to pneumonia as the journey length increased (5, 6, 27). In addition, all horses used in Experiment 1 shown in Table 1 were not confirmed healthy in respiratory organs by bronchoalveolar lavage (BAL), transtracheal wash (TTW) or ultrasound prior to departure (14). Although data from the present study were used to analyze body temperature variations during transportation, the factors mentioned above should be considered as limitations to this study.

While keeping these limitations in mind, analysis of the fluctuation in rectal temperatures during transportation indicated a tendency for the percentage of horses with pyrexia to increase after 20 h. A similar trend was observed previously (10, 14). However, the novel finding of the present study is that pyrexia arising during transport does not always persist to the end of the journey. Some horses develop pyrexia that persists to the end of transportation, while in other horses, the pyrexia resolves before the trip is over, and they appear clinically healthy at the time of arrival.

Another limitation to the study is that final assessments of horses were conducted at the conclusion of transit, meaning that horses with pyrexia during transit but not at the end of the journey, were not followed out for a designated time period. Hence it is impossible to draw conclusions about the clinical significance of spontaneously resolving pyrexia during transport.

At necropsy, many of the cases that exhibited antemortem pyrexia, were associated with acute sero-purulent bronchopneumonia. This suggests that either these lesions arose during transport, or as an exacerbation of pre-existing subclinical pulmonary pathology. It is especially noteworthy that the bacterial species isolated from pneumonic lesions were oral-pharyngeal commensal species, such as *Streptococcus* spp., which are normal flora of the upper respiratory tract. Environmental organisms, especially airborne, were not isolated from the lesions (4, 16). This kind of acute pneumonic lesion is probably caused by an elevated head position for an extended period of time over the course of a long journey (5, 6, 27). This position enables commensal bacteria to invade the lower airway when pulmonary clearance mechanisms are compromised, as shown by the presence of inhaled hay particles within alveolar lumen, causing lower airway contamination and endogenous opportunistic infection during transport (11). This increases opportunistic bacterial load, which is known to be a risk factor for shipping fever (5, 6, 11, 27). Once these lesions form during transport, differences in individuals' immune response or in the virulence of pathogens may determine which cases involve worsening inflammation and which recover, thereby leading to differences in body temperature upon arrival. Horses that exhibit normal temperature at the end of transportation and appear healthy could have subclinical pneumonia. In these horses, internal or external factors could cause pneumonic foci to deteriorate after arrival. It has been reported that pleuropneumonia usually becomes clinically evident in the week after transportation (18).

Lastly, although we investigated the possibility of using the arrival temperature of the horses to predict fever onset during transport, no significant results were obtained. Therefore, the AUC obtained from this search was low at 0.56. For reference, when judging prediction and diagnosis capabilities based on AUC values, an AUC of 0.9–1.0 indicates high accuracy of potential clinical predictors, 0.9–0.7 indicates moderate accuracy, and 0.5–0.7 indicates low accuracy (21–23). Using 38.6°C as the cut-off value for the temperature upon arrival to diagnose the presence or absence of transport-related pyrexia had a specificity of 89.3%, but a low sensitivity of 32.0%, and thus, using this cut-off value was not a statistically significant method to diagnose the presence or absence of transport-related pyrexia, and its diagnostic precision was denied (Table 5). However, there are many recent studies that compare the AUC of multivariate models created using multivariate logistic regression models and Cox proportional hazards models, instead of the univariable analysis we used in this study (28, 29). Therefore, by utilizing these multivariate analysis methods, a new diagnostic method may be developed in the future that could easily predict transport-related pyrexia by focusing on body temperature measurements before and after transportation.

## Conclusions

Early detection of horses at a high risk of shipping fever pneumonia is of utmost importance in maintaining healthy and successful athletic horse populations. Early and accurate detection of at risk individuals allows for focused prophylactic treatment and decreased losses to the sport horseAvailable online at: industry.

As such, the correlation between development of shipping fever during transportation and body temperature upon arrival was examined. Using 38.6°C as the cut-off value, the specificity of diagnosing the presence or absence of shipping fever was 89.3%. However, the sensitivity of this cut-off value was low, at 32.0%. With regard to both sensitivity and specificity, the temperature upon arrival has low diagnostic power, and it is not possible to accurately predict the presence of shipping fever based on this parameter. Therefore, the updated recommendation based on the data presented here is to take repeated rectal temperature measurements at regular intervals of no more than 6 h, after 20 h of transport.

## Data Availability Statement

All relevant data are within the paper. The raw data supporting the conclusions of this manuscript will be made available by the corresponding author to any qualified researcher.

## Author Contributions

MO contributed conception of the study, design, collection of samples and data, and wrote the manuscript. YM performed statistical analysis under supervision of MO.

### Conflict of Interest Statement

The authors declare that the research was conducted in the absence of any commercial or financial relationships that could be construed as a potential conflict of interest.
